# Tilted Spins in Chains of Molecular Switches on Pb(100)

**DOI:** 10.1021/acsnano.4c07477

**Published:** 2024-09-14

**Authors:** Marten Treichel, Jenny Möller, Xiangzhi Meng, Florian Gutzeit, Rainer Herges, Richard Berndt, Alexander Weismann

**Affiliations:** † Institut für Experimentelle und Angewandte Physik, Christian-Albrechts-Universität zu Kiel, 24098 Kiel, Germany; ‡ Otto-Diels-Institut für Organische Chemie, Christian-Albrechts-Universität zu Kiel, 24098 Kiel, Germany

**Keywords:** spin crossover molecules, spin excitations, magnetic anisotropy, spin canting, molecular switch, surface facetting

## Abstract

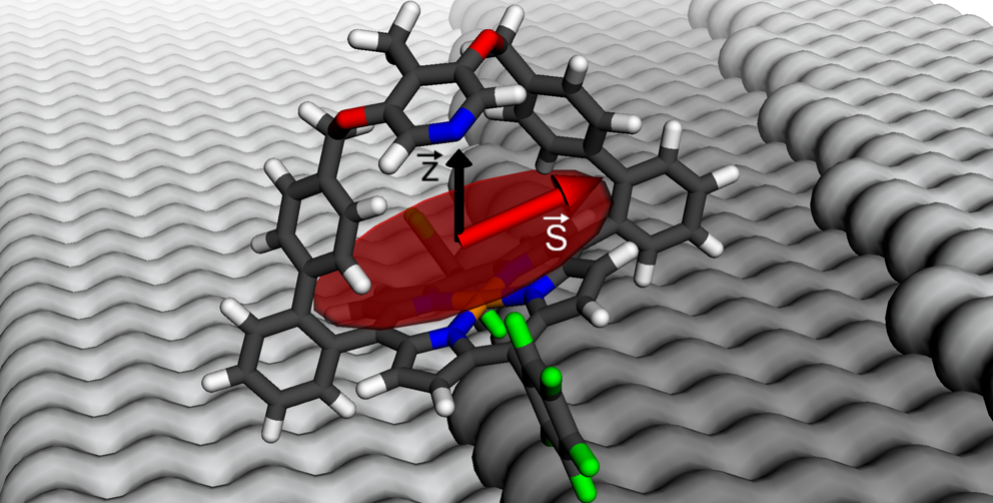

A complex based on a Ni(II) porphyrin exhibiting spin crossover on Ag(111) is studied on Pb(100) by scanning tunneling microscopy at 0.3 K. Strong molecular interactions between the phenyl and pentafluorophenyl moieties lead to the formation of molecular chains and cause a faceting of the substrate surface. The chains are located along double and multiple substrate steps that deviate from high-symmetry directions. Tunneling spectroscopy reveals spin-flip excitations of an *S* = 1 system. Measurements in high magnetic fields are used to identify a tilt of the complex and its hard anisotropy axis with respect to the surface normal. Electron injection into the substrate near the molecular rows induces a transition to a state with larger inelastic cross section, leaving the spin state unchanged.

Magnetic molecules may find applications in data storage, displays, and sensors.^1−4^ The interaction of such molecules with conducting substrates has not been widely explored and is likely to be relevant.^5,6^ In particular, the magnetic properties of spin crossover (SCO) complexes can be switched between different spin states using stimuli such as light and electric current.^7,8^ Concerning SCO molecules adsorbed on surfaces, current-induced bistability has been repeatedly reported. Spin state switching has been identified from X-ray adsorption spectroscopy,^9−16^ the observation of zero-bias anomalies in STS,^9,11,13,17^ modifications of the molecule’s apparent height,^11,13,17−20^ changes of frontier orbitals and vibration intensities,^13,21,22^ and the observation of current fluctuations.^19,21−26^ In addition, control experiments with analogous but magnetically passive compounds have been used to infer spin switching.^24,25^

Here, the spin-crossover complex HCP (hairclip-porphyrin, 5,15-(2′-(4″-(4‴-methylpyridin-3‴,5‴-ylen)-oxymethyl-phenyl)phenyl)-10,20-bis(2,3,4,5,6-pentafluorophenyl)-Ni(II)porphyrin) is investigated. It has been designed to achieve two stable spin states via geometrical changes of a strap containing a pyridine moiety (Figure 1a). Depending on the proximity of the pyridine to the Ni atom, spin states *S* = 0 (large separation) or *S* = 1 (coordinative bond) are obtained. This functionality also relies on a coupling between the spin and the structure of the porphyrin platform (ruffled or planar, respectively). It has been shown that this complex undergoes current-induced spin-state switching on Ag(111).^13^ By comparison of orbital energies of single molecules measured with a scanning tunneling microscope (STM) and those determined from powder samples of the compound, which are known to exhibit SCO, the switching was attributed to the above-mentioned transition between *S* = 0 and 1.

**Figure 1:**
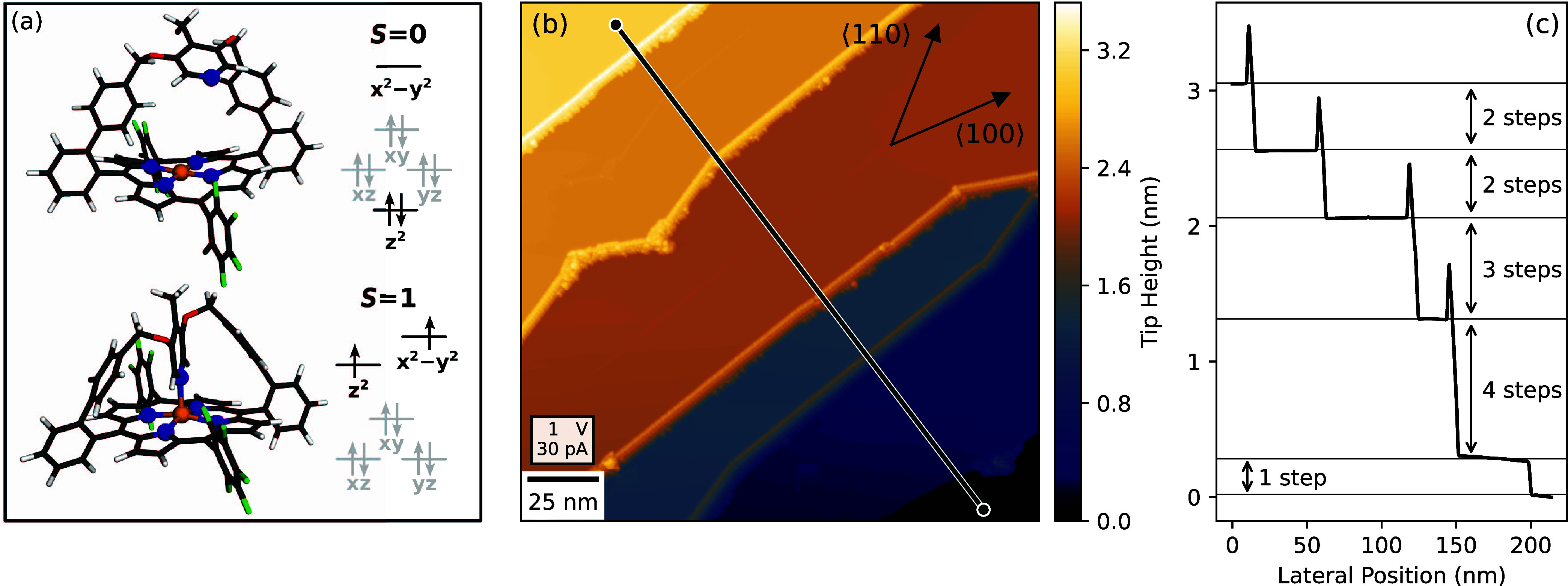
(a) Models of the complex HCP. C, H, F, and O atoms are shown as black, gray, green, and red sticks, respectively. N and Ni atoms are shown as blue and brown spheres. Depending on the spin state of the complex, the strap containing a single N atom is close to the Ni atom of the porphyrin platform (*S* = 1, lower scheme) or at larger distance (*S* = 0, upper scheme). *d*-level occupations are schematically indicated. (b) Overview constant-current image (180 × 180 nm^2^) of Pb(100) after exposure to a submonolayer amount of HCP. Straight step edges are observed that deviate from high-symmetry directions and are decorated with molecules. (c) Cross-sectional profile along the black line in (b). Single steps (height 248 pm) are rarely observed and free of molecules. Molecules appear as sharp overshoots in the profile and are only found at double, triple, and multiple steps. *T* = 4.2 K.

In the present work, a superconducting Pb(100) substrate was used motivated by the possibility that a localized molecular spin can lead to the formation of sharp spectral features in the superconducting gap, the so-called Yu-Shiba-Rusinov resonances,^27−30^ which in turn can serve as a direct fingerprint of the spin. However, tunneling spectroscopy at mK temperatures and in magnetic fields from 0 to 9 T rather revealed spin excitations^31−41^ of an unscreened spin *S* = 1. We find a magnetic anisotropy with the hard axis being tilted away from the substrate normal. In addition, the complex induces faceting and arranges itself into an intriguing ordered pattern at double and multiple substrate steps. We propose a geometric model of tilted molecules at steps that is consistent with the observed orientation of the hard axis. Current injection into the substrate induces an irreversible transition to a new state, but the zero-field spin excitation energy remains unchanged. We tentatively attribute the absence of magnetic bistability to a surface *trans* effect,^42^ where bonding to substrate steps fixes the spin state and reduces the influence of the opposite pyridine ligand.

## Results and Discussion

Figure 1b presents an overview of a typical Pb(100) surface area after exposure to a submonolayer amount of HCP. We find fairly wide terraces with widths exceeding 50 nm. Stable imaging of HCP on flat terraces was not achieved. Molecules are stably observed at multiple steps while terraces and single steps (height 248 pm) appear to be free of HCP as illustrated by a cross-sectional profile across several steps (Figure 1c). The number of parallel molecular rows is found to be identical to the number of atomic steps.

The decorated step edges exhibit characteristic orientations of ±15° with respect to the ⟨100⟩ and ⟨110⟩ directions of the substrate lattice. These orientations result in angles between straight sections of the steps of 135, 150, and 165° (Supporting Information, Figure S1).

The preponderance of multiple steps with their specific orientations and the large lateral extent of the planar areas deviate from the appearance of our pristine Pb(100) substrate, which exhibits fairly small terraces (typically a few nanometers wide). Apparently, the adsorption of HCP induces a pronounced faceting and step bunching of the crystal surface.^43,44^

**Figure 2:**
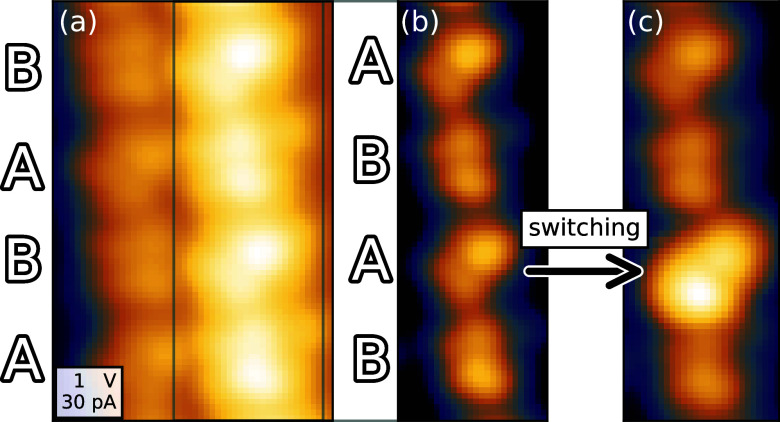
HCP at steps: orientation and switching. (a) Topograph of a molecular double chain. Two orientations labeled A and B are resolved. (b) Topograph of the upper row with adjusted contrast. (c) The same area imaged after injecting current into the Pb substrate close to the imaged area. One of the A molecules has been converted to a new state A* (arrow) that appears ≈140 pm higher.

At double and multiple steps, HCP molecules appear as a pair of lobes with slightly different apparent heights. Two orientations of this dumbbell pattern are observed and labeled A and B in Figure 2. They exhibit maximum apparent heights with respect to the upper Pb terrace of 520 ± 10 and 495 ± 10 pm (at *V* = 1 V), respectively. We find intermolecular distances of 1.35 ± 0.05 (1.50 ± 0.1) nm parallel (perpendicular) to the step edges. Types A and B usually alternate along a chain although type B molecules occasionally are nearest neighbors while A molecules are hardly found next to each other. By removing a molecule from a double row with the STM tip, the position of the molecules relative to the step underneath may be estimated (Supporting Information, Figure S4).

### Current-Induced Modification

Attempts to switch HCP to a different state by applying elevated voltages or currents with the STM tip directly above the molecule failed and usually led to largely disordered areas in the molecular chains, tip modifications and instability. However, transitions were reproducibly achieved by placing the STM tip at a lateral distance of ≈10 nm from a molecular row and injecting current, typically at *V* = 3 V and *I* = 100 pA over a period of 30 s or more (Figure 2). The process was not reversible. Contrast between pristine and modified molecules is clearly observed *V* = 1 V but vanishes at low bias (<200 mV). Interestingly, only the type A molecules in the upper row of the molecular double or multiple rows could be manipulated in this manner. Occasionally, molecules in the second topmost row of wider chains were also switched. A single manipulation attempt resulted in up to 5 molecules that were switched within a 15 nm section of the nearest chain. The switched molecules appear ≈140 ± 20 pm higher than in their pristine state (Supporting Information, Figure S5) and remained stable over hours. Whether the different appearances in STM images indicate different spin states will be discussed below.

### Low-Bias Spectra

Next, we explored the spectroscopic properties of the molecules, with both the Pb(100) surface and the STM tip in the superconducting state. To this end, we measured *I*(*V*) spectra on a dense grid of lateral positions and numerically calculated the differential conductance d*I*/d*V*. The tip height was set by first imaging the surface at low current (≈5 pA) and then bringing the tip ≈250 pm closer to the sample at each grid point. This mode of measurement proved to be less prone to inducing instabilities of the tunneling junction compared to slower measurements using a phase-sensitive detection scheme or imaging at higher current.

**Figure 3:**
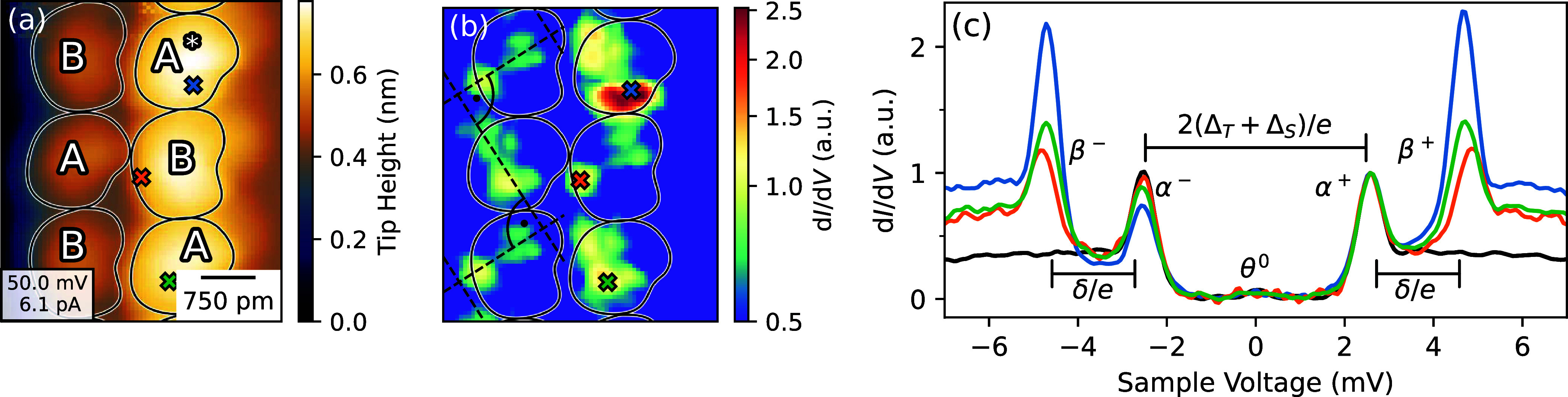
Low-bias spectra of A, B, and A* molecules (*T* = 4.2 K). (a) Topograph recorded at 50 mV and 6 pA. The apparent heights of A and A* are almost identical at this voltage. Black contours around the molecules serve to guide the eye. (b) Map of d*I*/d*V* at *V* = 4.72 mV. Each molecule exhibits a dumbbell shaped pattern. The patterns of A and B are rotated by 90° with respect to each other. The differential conductance of A* is significantly larger. (c) Spectra of A, B, and A* (green, orange, blue) recorded at the positions indicated by crosses in (a, b). Two pairs of peaks, each symmetric around the Fermi level (*V* = 0) are resolved. We attribute them to coherence peaks (α^+^, α^–^) and spin excitations (β^+^, β^–^). The excitation energy δ may be determined from the separation of the α and β peaks as indicated by horizontal markers. A weak feature (θ^0^) due to tunneling of thermally excited carriers is present around *V* = 0. A spectrum taken on the free substrate is shown in black.

Figure 3 shows the results. Panel (a) presents a topograph recorded at 50 mV and 5.6 pA. A and B type molecule are labeled in the image. The molecule labeled A* was previously manipulated as described above. At the voltage used, it cannot be distinguished from A molecules in the topograph. However, the d*I*/d*V* map measured at *V* = 4.7 mV (Figure 3b) provides clear contrast between A, A*, and B molecules. The conductance map reproduces the alternating molecular orientations along the chains. In particular, the A and B molecules show very similar dumbbell-shaped patterns that are perpendicular to each other (black dashed lines). Interestingly, the orientations of the dumbbell patterns do not alternate symmetrically with respect to the chain direction but are rather found at ≈−30° and ≈+60°, respectively, which is ≈15° larger than ±45°. These data suggest that types A and B are indeed identical molecules as expected, but are differently orientated on the surface.

Characteristic d*I*/d*V* spectra of the three molecular states A, A*, and B are shown in Figure 3c. They were recorded at the positions indicated by crosses in matching colors in the topograph and have been normalized to identical heights of the peak labeled α^+^. The spectral features of all states are fairly similar and comprise coherence peaks (α^+^, α^–^) separated by the combined energy gap of the tip and the sample, a weak signal close to zero bias (θ^0^) that is due to tunneling of thermally excited quasiparticles, and two intense features (β^+^, β^–^). Energy diagrams for all transitions are displayed in the Supporting Information, Figure S11. As will be shown below, the β peaks are due to spin excitations.

The map of Figure 3b presents the spatial distribution of β^+^, i.e. the spin excitation signal. The inelastic excitation probability exhibits intramolecular contrast and is particularly high above the switched molecules (A*). Spectra of the molecules (Figure 3c) confirm this observation. β peaks of A* can be up to 2.5 times higher than the coherence peaks α while the ratio is close to 1 on A and B molecules. Furthermore, the d*I*/d*V* signal of A* (blue curve) displays a small asymmetry of the coherence peak height and suggests that a Yu-Shiba-Rusinov resonance may be present albeit not separately resolved.^45^

### B-Field Dependence

**Figure 4:**
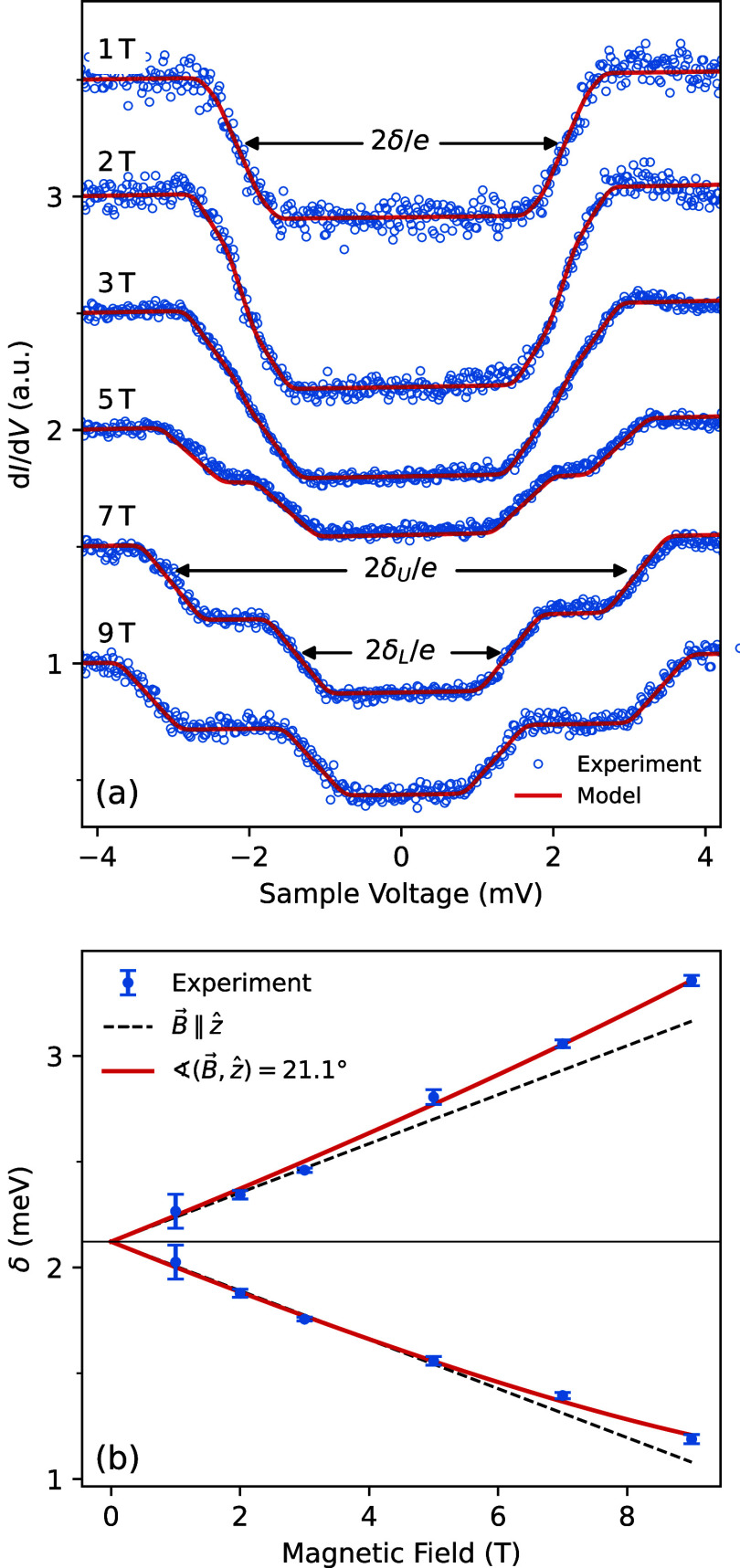
(a) d*I*/d*V* spectra (blue circles) of A* molecules in magnetic fields (indicated at each spectrum) along the surface normal (*T* = 330 mK). Superconductivity is suppressed. The spectra are vertically shifted by 0.5. At elevated fields, the steps separated by 2δ/*e* split into two steps separated by 2δ_L_/*e* and 2δ_U_/*e*. Fits discussed in the text are shown by red lines. (b) Magnetic field dependence of the excitation energies δ determined from the fits in (a). Estimated uncertainties of δ are indicated. Dashed lines show a fit using eq 3 within second order^46^ assuming *B⃗* to be parallel to the spin anisotropy axis. Better agreement is obtained when a tilt of ≈21° is used (solid lines).

To determine the origin of the spectral features β, further measurements were performed with a magnetic field applied along the surface normal. Figure 4 shows the evolution of the experimental spectra (blue circles) with the strength of the field. The spectra were acquired on slightly different lateral positions and the signal amplitudes should therefore not be directly compared. At low field (1 T) steps are observed symmetrically around zero bias and approximately at ± δ/*e*, i.e., the separation between β and α peaks in Figure 3c. The α structures are absent because the tip and the sample are in a normal conducting state at elevated *B*-fields. The steps broaden at larger magnetic field, are split into double steps at 5 T, and move to larger |*V*| as the field is further increased. Because the amplitudes of the inner and outer steps are identical, these data suggest that the steps are due to the excitations of an *S* = 1 system from *m*_S_ = 0 to ±1. To more precisely determine the excitation energies, we fitted the data using temperature-broadened step functions^46^
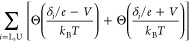
1where *k*_B_ is the Boltzmann constant and
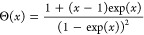
2The lower (δ_L_) and upper (δ_U_) excitation energies, the effective temperature *T* describing the width of the steps, the step amplitude, and a linear background were adjusted. More details may be found in the Supporting Information. The model provides fairly good fits (red curves) of the data.

The excitation energies from the fits are displayed in Figure 4b, which reveals shifts toward lower and higher values for the lower (blue points) and upper (orange points) step, respectively. The deviations of this Zeeman splitting from linearity (dashed lines, calculated for *g* = 2) suggest that the orientation of the spin anisotropy axis does not match the direction of the magnetic field. We model this scenario using the following Hamiltonian
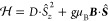
3where the direction of ***B*** = (*B*_*x*_, *B*_*y*_, *B*_*z*_) = *B*(sin(ϕ), 0, cos(ϕ)) deviates by ϕ from the spin anisotropy axes (*z* direction). ***Ŝ*** describes the spin matrices for *S* = 1, *g* is the Landé factor, and μ_B_ denotes the Bohr magneton. The first term describes zero-field splitting via an uniaxial magnetic anisotropy *D* and the second term represents the Zeeman splitting.

Fits of the eigenvalues of this Hamiltonian, which are analytically derived in the Supporting Information, match the experimental data well (Figure 4b, solid lines). We find a positive *D* = 2.12 meV, i.e. an easy-plane anisotropy, a tilt of the spin anisotropy axis of ϕ = 21.1 ± 1.0° from the surface normal, and *g* = 2.26 ± 0.03. In the experiment, *B* is perpendicular to the substrate surface. The measured tilt angle therefore suggests a tilt of ≈21° between the molecular macrocycle and the Pb(100) substrate.

It should be noted that the close proximity of a STM tip typically reduces *D*.^27,35,47,48^ Comparing the apparent height of HCP in our STM images with the geometric height of the gas-phase structure we estimate that the distance between the tip and the topmost atom is 600–800 pm smaller above the molecule than on the metal substrate. The tip thus comes very close to the molecule. This likely is the reason for the difficulties incurred in imaging and also implies that the value 2.12 meV should be regarded as a lower limit of *D*. In fact, the β peak positions in spectra from different positions vary by up to ±0.3 meV, presumably because of varying perturbation by the tip.

Substitution of the oxygen atoms of HCP by sulfur leads to a closely related compound. In powder form, this molecule is in an *S* = 1 state and does not exhibit spin state transitions under illumination or as a function of temperature.^49^ From the temperature dependence of its susceptibility, an anisotropy energy of 2.23 meV and *g* = 2.16 have been determined. These values are astonishingly close to our results for HCP at Pb(100) steps (*D* = 2.12 meV and *g* = 2.26).

Close inspection of Figure 4a reveals small, systematic deviations of the fits from the data. However, the perturbative scattering calculations underlying the model of eq 1 may be extended to third order terms.^46^ As shown in Figure S12 of the Supporting Information, the overshoots above the step edges, in particular above the low-energy steps near ±2 mV, are well reproduced by the extended model. From this fit the Kondo coupling *J*ρ_s_ can be estimated. Together with the magnetic anisotropy *D* and the gap parameter Δ it determines whether a high-spin molecule on a superconductor shows YSR states, spin excitations, or both.^35,50^ As expected, the fit is greatly improved when the tilt is taken into account.

The above discussion revealed that the β peaks are due to spin-excitations of a *S* = 1 complex with easy-plane anisotropy. Returning to the data of Figure 3c, we recall that the spectra of A and A* are qualitatively identical. Consequently, the switching observed on Pb(100) cannot be due to a change of the spin state from *S* = 0 to 1 or vice versa. This leaves a geometrical bistability of the adsorbed complex as the most likely interpretation. On the other hand, spin switching has been reported from the same complex on Ag(111)^13^ and the changes of the topographs upon switching were almost identical to those in the present case. Considering that the STM is predominantly sensitive to the geometrically highest features of the molecule we hint that the strap conformations changes just like in the free molecule, namely between a coordinative N–Ni bond and a large N–Ni distance. However, the spin state of the molecules at Pb steps is always *S* = 1, independent of the N position. In other words, the stepped Pb surface appears to act as a fifth ligand to the Ni ion and promotes it to *S* = 1 from its gas-phase, low-spin state. This scenario is inspired by the surface *trans* effect reported from other transition metal complexes, where bonding to the substrate occurs and weakens the bond of the opposite ligand.^42^

The absence of a spin-state change on Pb(100) does not imply that HCP does not exhibit spin switching on other substrates. In fact, the earlier work on Ag(111) provided ample evidence of a change of the spin state from X-ray magnetic circular dichroism (XMCD) experiments, the presence/absence of a Kondo feature in the two states, and a shift of highest occupied molecular orbital (HOMO) energies in agreement with density functional theory (DFT).^13^ On Pb(100), the HOMO could not be spectroscopically resolved because instabilities occurred at the required negative sample voltages. As detailed in the Supporting Information (Figures S9 and S10), the lowest unoccupied molecular orbital (LUMO) energy of the molecular state A is ≈500 meV lower than on Ag(111) and is further reduced for A*. There are other important differences between Pb(100) and Ag(111) as can be seen from the different growth modes (chains at steps on Pb(100) vs two-dimensional, flat islands on Ag(111)) and the reversibility of the switching on Ag(111), which was not found on Pb(100).

## Structure Model

**Figure 5:**
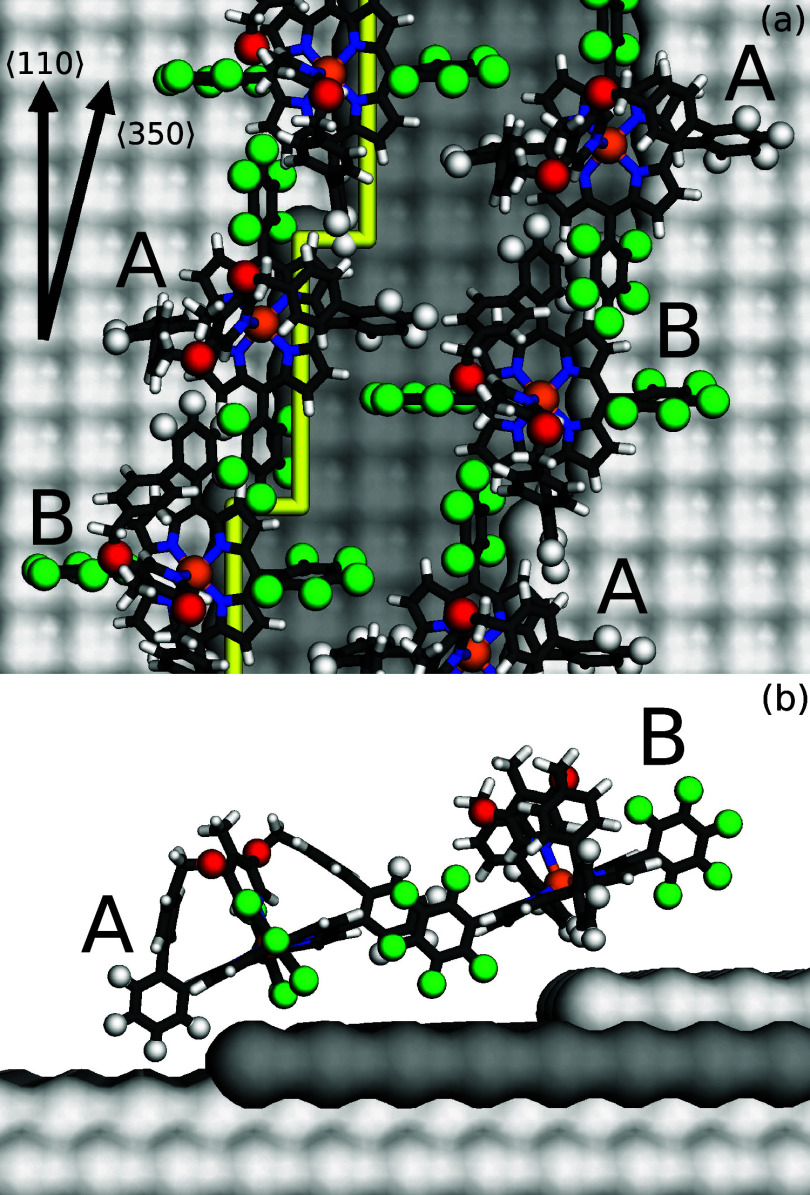
Proposed model of the adsorption structure of HCP at double steps. (a) Top view. The upper (right) and lowest (left) Pb terraces are displayed as light gray atomic patterns. The middle terrace is 4.5 atoms wide and colored gray. The steps run along a crystallographic ⟨350⟩ directions and are comprised of segments of 4 atoms along a ⟨110⟩ direction and kinks. The substrate structure has been blurred to enhance the visibility of the atoms of the molecules. A yellow line highlights one of the steps. C, H, N, and Ni atoms are shown as black, white, blue, and orange sticks, respectively. F and O atoms are displayed as green and red spheres. The optimized structure from gas-phase calculations (*S* = 1) is used. The position of the molecules along the ⟨350⟩-direction and their distance from the surface were arbitrarily chosen because the experimental data do not provide direct information on these parameters. (b) Side view of two molecules and the substrate.

Putting together the above information, we tentatively propose a structure model of a molecular double chain and the substrate steps underneath (Figure 5). It was derived using the gas-phase geometry of the molecules as obtained from DFT calculations and is consistent with the STM images of double chains. In particular, the intermolecular distances and the step orientation in the model closely match the measured values. Along each chain, adjacent molecule are rotated by 90° with respect to each other. This pattern reflects the observation that A and B molecules exhibit identical spectral signatures. The arrangement further enables a parallel orientation of the phenyl (white) and pentafluorophenyl (green) subunits of neighboring molecules along the chain. This binding mechanism is known^51−54^ and interconnects parallel chains. This bonding scheme appears to be the cause of the particular step orientations found.

An important difference between alternating molecules along the chain and opposite molecule of double chains can be observed. In half of the molecules, both phenyl subunits bind to adjacent pentafluorophenyls, in the other half one phenyl remains free to move. Considering that the geometric changes between the low and high spin states of the complex involve a significant conformational change of the phenyl, the bonding scheme provides an explanation for the presence and absence of switchability of the A and B complexes, respectively. The molecules in the model of Figure 5 are labeled accordingly. The fact that a few A molecules in the second topmost row of wider chains were converted into A* indicates that the steric hindrance perpendicular to the chain direction is not complete.

The plane defined by the stairway in Figure 5 is tilted by approximately 10° with respect to the (100) surface. On the other hand, measurements of the magnetic anisotropy indicate an angle of ≈20° between the porphyrin plane and the substrate. This factor-two difference is qualitatively consistent with the proposed geometry.

While the proposed structure reflects the ≈90° rotation of the patterns of adjacent molecules in low-bias conductance data (Figure 3b) it seems inconsistent with the contrast in topographs measured at 1 V (Figure 2). Here, alternating molecules seem rotated by ≈45° and, in addition, show different relative heights of the lobes. We attribute these complications of the image contrast to a combination of the tilted adsorption geometry at steps and the distribution of the electronic states over the bridge-part of the complex. Unfortunately, the size and complexity of the molecule–substrate system precludes reliable ab initio calculations to verify this hypothesis.

## Comparison with X-ray Crystal Structure

The crystal structure of HCP determined using X-ray diffraction^49^ is inconsistent with the structures found on Pb(100). In molecular single crystals of HCP neighboring molecules are found with their strap pointing in opposite directions. In contrast the related molecule NiTPPF_10_ (5,15-bisphenyl-10,20-bis(2,3,4,5,6-pentafluorophenyl)-Ni(II)porphyrin), which does not contain the strap, shows a crystal structure that closely matches the assembly pattern found on Pb(100).^49^ Parallel to the porphyrin plane, the molecules form a square lattice with molecule–molecule distances of 15.3 Å and the angle between the lattice vector connecting the Ni-atoms of adjacent molecules and the platform orientation (defined by two opposite meso-positions of the porphyrin) is 13.6°. This angle is very similar to the orientation of the molecular chains with respect to the ⟨100⟩ and ⟨110⟩ directions and suggests that the (pentafluoro-) phenyl groups tend to align parallel to a high symmetry direction. The crystal structure of NiTPPF_10_ from X-ray diffraction overlaid to a STM image of HCP is presented in the Supporting Information, Figure S6, and matches the proposed structure in Figure 5. A control experiment with NiTPPF_10_ on Pb(100) showed similar surface faceting and molecular chains at double and multiple substrate steps as described above. In addition, isolated islands on the flat surface were found (Supporting Information, Figure S7).

## Conclusions

The spin-crossover complex HCP induces faceting of the Pb(100) surface. The molecules arrange themselves in double and multiple rows that ride on double and multiple substrate steps. The step orientations deviate from the high-symmetry directions of the substrate and appear to be imposed by the molecules that interact via phenyl-pentafluorophenyl pairs. Irreversible modification of the molecules may be induced over a nm distance by injecting hot electrons into the substrate. The resulting changes of STM topographs are not due to a spin transition but may be qualitatively understood in terms of a geometrical change of the molecule. Detailed measurements in a magnetic field reveal a high-spin, *S* = 1 state with an easy-plane anisotropy of *D* ≈ 2.1 meV and a *g*-factor of 2.26, i.e. values that are very similar to powder data. The hard axis tilted away from the surface normal by ≈21°.

The properties of HCP on the Pb(100) surface are substantially different compared to a Ag(111) substrate. This includes the orbital energies and the impact of the adsorbed molecules on the substrates themselves. On Pb(100), certain HCP molecules can be prepared in two states that are stable over hours. While the STM image contrast of these states is very similar on both substrates, a bistability of the spin states does not occur on Pb, in contrast to the case of Ag(111).^13^ We tentatively interpret these observations in terms of a surface *trans* effect^42^ that reduces the impact of the axial pyridin ligand on the spin state. This is consistent with earlier work on adsorbed transition metal complexes and small axial ligands like NO or NH_3_ that revealed the key-role of the substrate as a additional ligand.^55−57^ In the present case, the molecules are not inert either but significantly affect the substrate structure.

## Methods

### Synthesis

HCP and NiTPPF_10_ were synthesized according to the description of ref (49).

### Sample and Tip Preparation

Ar ion bombardment and annealing to 270 °C were used to prepare Pb(100) surfaces. HCP was sublimated from a crucible heated to (≈350 °C). STM tips were cut from Pb wire and sputtered *in vacuo*. Experiments were carried out using a low temperature STM operated in ultrahigh vacuum (Unisoku USM1300).

### STM

Topographs were measured in the constant-current mode of operation. Stable imaging of the molecules required a fairly low current (30 pA) and slightly elevated voltage (1 V). d*I*/d*V* spectra were obtained by numerical derivation of current–voltage characteristics, followed by a convolution with a moving average and a Savitzky–Golay filter.^58^

## Supporting Information Available

STM Data; LUMO spectra; energy diagrams and analytical solutions of the characteristic equation (PDF)
