# Supplementary material for: Tilted Spins in Chains of Molecular Switches on Pb(100)
Source: ACS Nano. 2024 Sep 14;18(38):26184–91. doi: 10.1021/acsnano.4c07477 (PMC11440647; doi:10.1021/acsnano.4c07477)
Supplement: Supplementary file 1 — nn4c07477_si_001.pdf [file nn4c07477_si_001.pdf]

Supporting Information to Tilted Spins in Chains of Molecular Switches on Pb(100)

Marten Treichel,† Jenny Möller,‡ Xiangzhi Meng,† Florian Gutzeit,‡ Rainer Herges,‡ Richard Berndt,† and Alexander Weismann*,†

†*Institut für Experimentelle und Angewandte Physik, Christian-Albrechts-Universität zu Kiel, 24098 Kiel, Germany*

‡*Otto-Diels-Institut für Organische Chemie, Christian-Albrechts-Universität zu Kiel, 24098 Kiel, Germany*

E-mail: weismann@physik.uni-kiel.de

Step Orientations

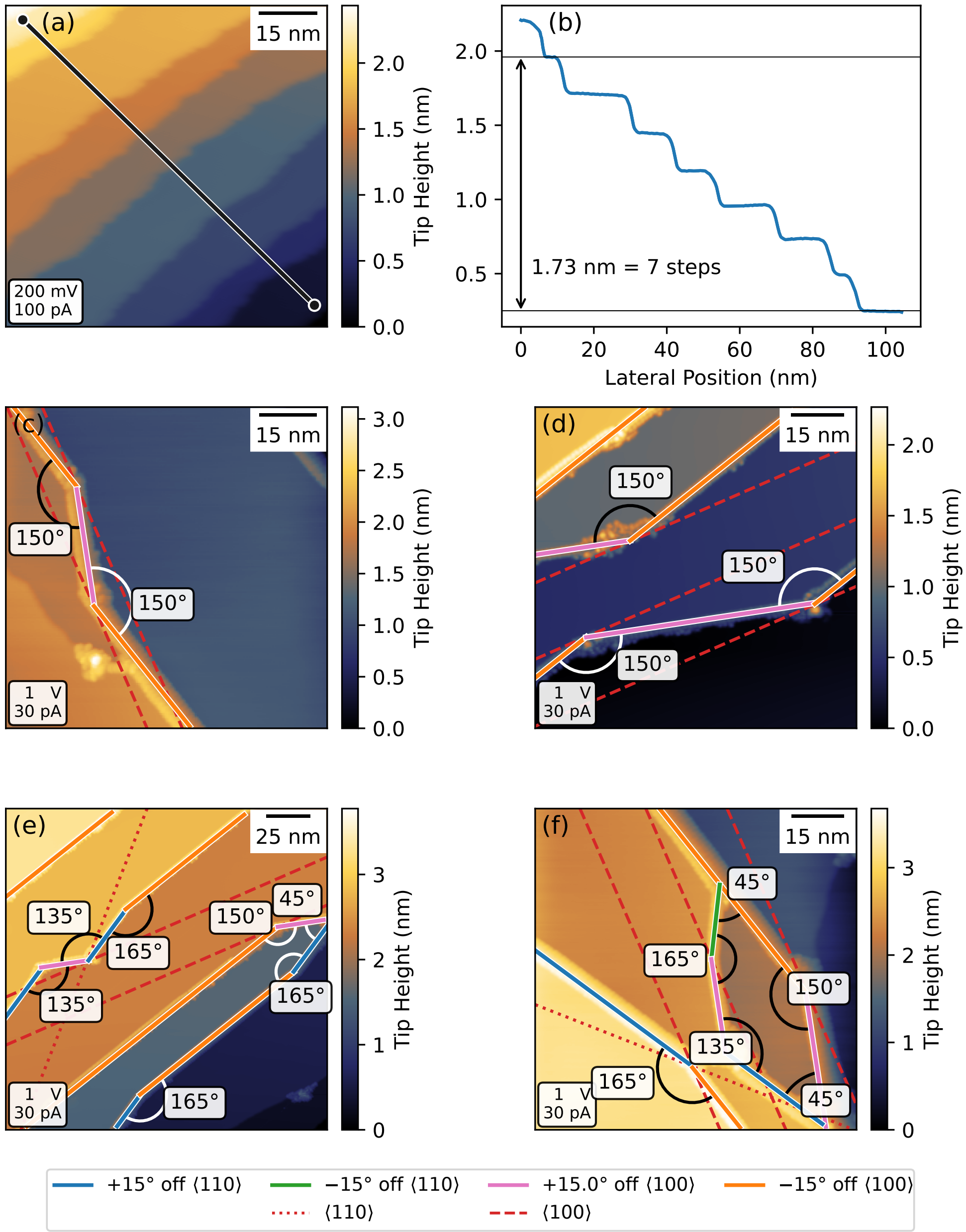

Figure S1: Overview constant-current images of Pb(100) (a) before and (c)–(f) after exposure to a sub-monolayer amount of HCP. A line-profile along the black line in (a) is displayed in (b) and shows only single substrate steps. In contrast, steps occuring in bunches of 2 to 4 single steps are covered with molecules in (c)–(f) while single steps are free of molecules. Dashed and dotted lines indicate crystallographic ⟨100⟩ and ⟨110⟩ directions. The molecular chains along ⟨140⟩ directions ($\approx \pm 15°$ with respect to ⟨100⟩) are marked by blue/green lines. Orange/pink lines mark chains along ⟨350⟩ directions *i. e.* at $\approx \mp 15°$ with respect to ⟨110⟩.

STM images of more switching events

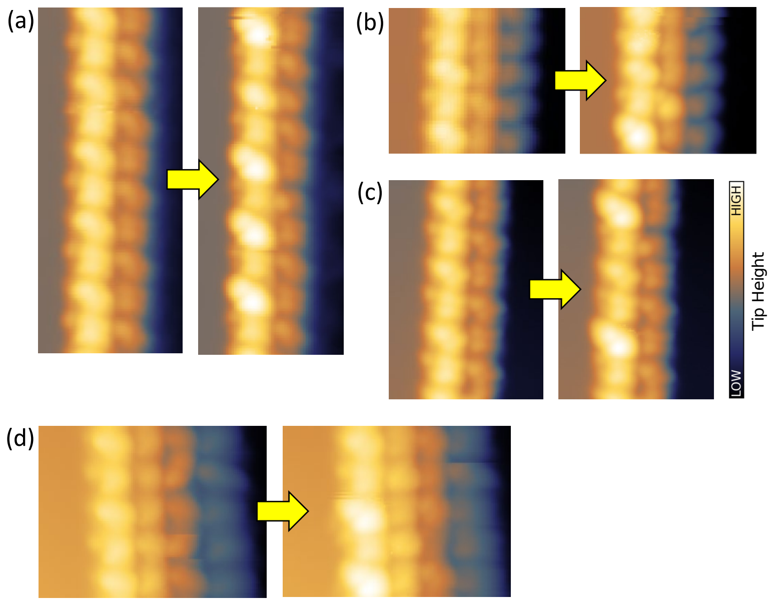

Figure S2: (a)–(d): STM topographs (1 V, 30 pA) before (left) and after (right) the application raising the sample voltage to 3 V at I = 100 pA in ≈ 10 nm distance from the chain on the upper substrate terrace. The colorscale spans 1.05 nm (a), 1.34 nm (b), 1.12 nm (c) and 1.74 nm (d). Panel (b) shows a rare event, where also a type A molecule on the second topmost molecular row is converted to A*.

STM images at larger coverages

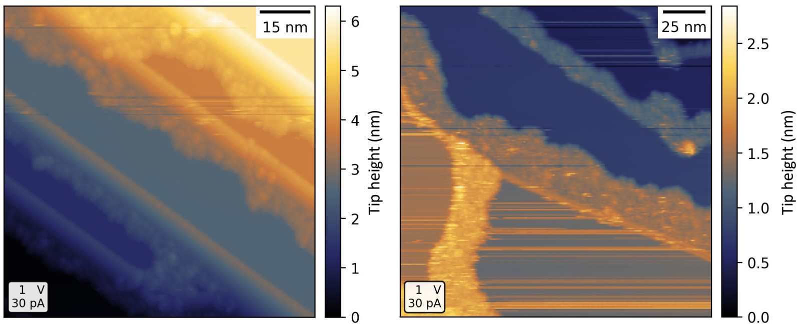

Figure S3: STM topographs of the Pb(100) surface at larger coverages of HCP. In addition to the molecular chains at bunched substrate steps, larger disordered areas can be observed.

Molecule Removal from Step

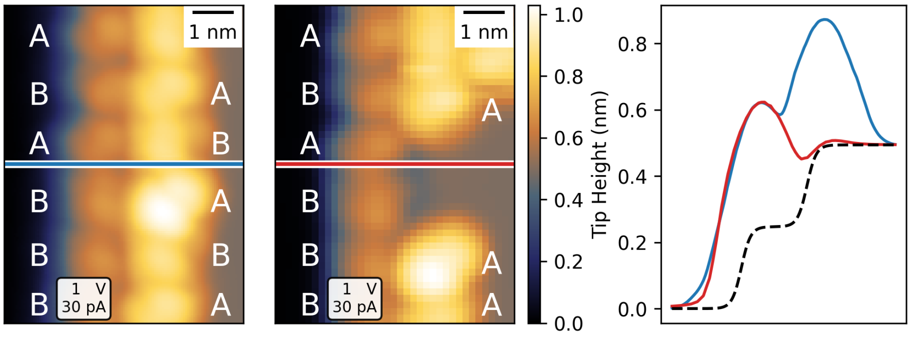

Figure S4: Topographs of a double step recorded before (left image) and after (right image) removing an A type molecule from the upper step with the STM tip. Cross-sectional profiles along the horizontal blue and red lines are displayed in the plot on the left hand side. The indentation of the profile recorded after the manipulation suggests that the removed molecule previously extended over two terraces. The dashed line shows an estimated profile of the Pb steps.

Profile of A* Molecules

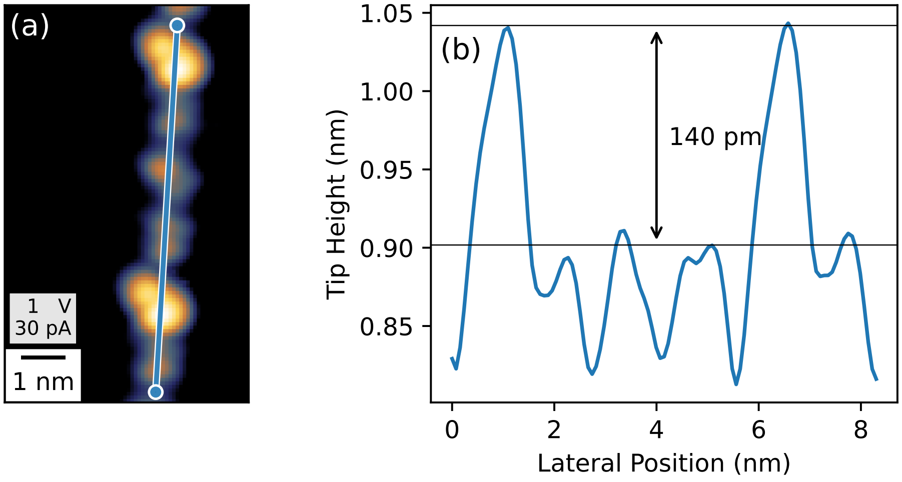

Figure S5: (a) Topograph of a molecule-covered double step. The contrast has been adjusted to show the upper molecular row in detail while the lower row is invisible. Two A molecules had been converted to A* before recording the image. They appear approximately 140 pm higher as show in the profile in (b) that was taken along the blue line in (a).

Comparison with the x-ray crystal structure of $NiTPPF_{10}$

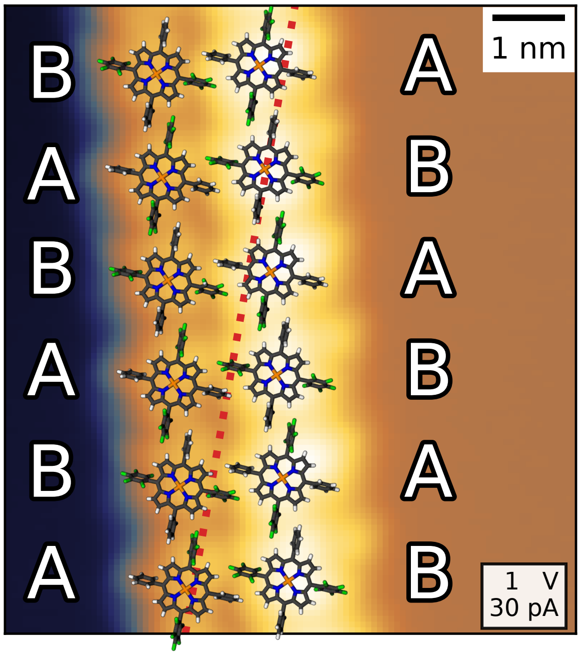

Figure S6: STM Topograph of a molecular double-row overlaid with the crystal structure of $NiTPPF_{10}$ from x-ray diffraction.[1] The pentaflourophenyl and phenyl rings are aligned with the $\langle 110 \rangle$ direction (red dots) of the substrate.

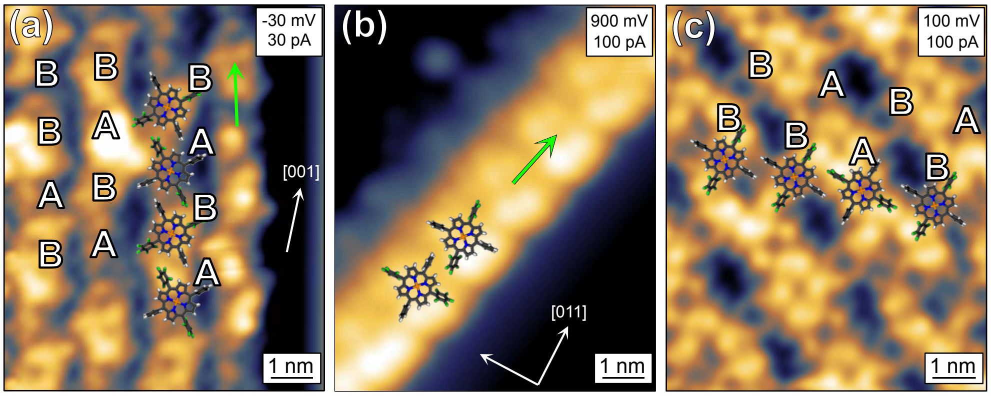

Figure S7: Self-assembly of $NiTPPF_{10}$ molecules (without bridge groups) on Pb(100). (a) Topograph of $NiTPPF_{10}$ molecules adsorbed over substrate steps. The orientation of molecular rows, indicated by a green arrow, forms an angle of $\approx 15°$ with the [001] direction of the surface. An alternating arrangement of A and B Molecules is also observed. (b) A second orientation of a molecular chain at substrate steps at an angle of $\approx 15°$ with respect to the [011]-direction of the substrate. (c) STM topograph of a molecular island on a flat surface area, showing a similar molecular arrangement as observed at substrate steps. The orientations of the substrate lattice in (c) and (b) are identical. The image in (a) has been rotated by 36° for clarity.

Model of a molecular chain containing an A* molecule

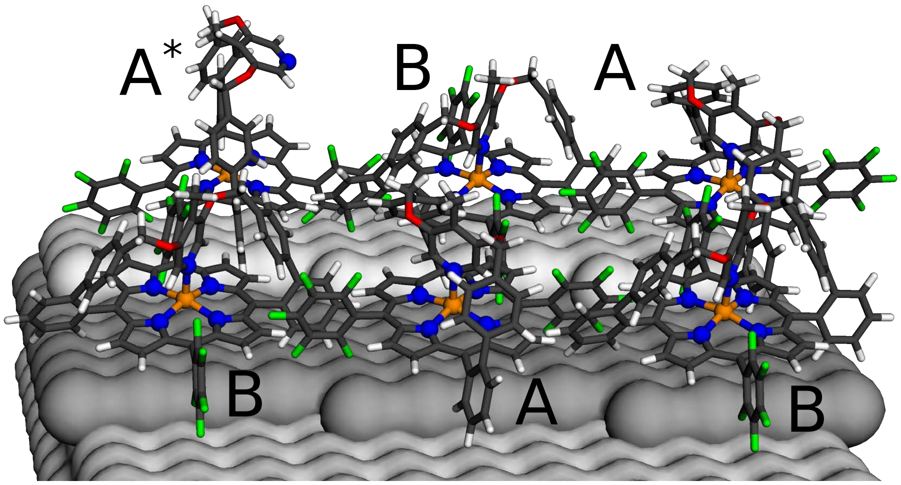

Figure S8: Three-dimensional representation of the model structure containing a switched A* molecule.

LUMO spectra

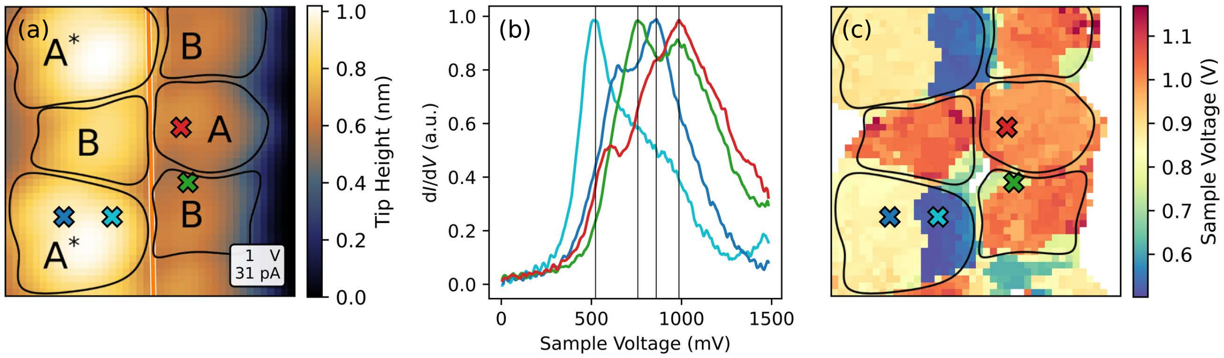

Figure S9: (a) Constant current topograph of a molecular chain containing two switched A* molecules (4.1×4.1 nm^2). (b) dI/dV-Spectra acquired at the positions marked in (a) and normalized to identical peak amplitudes. (c) Map of the voltage of the peak in dI/dV extracted from a grid of spectra. Spectroscopy at larger negative voltages resulted in modifications of tip and molecule so that the highest occupied molecular orbital could not be resolved.

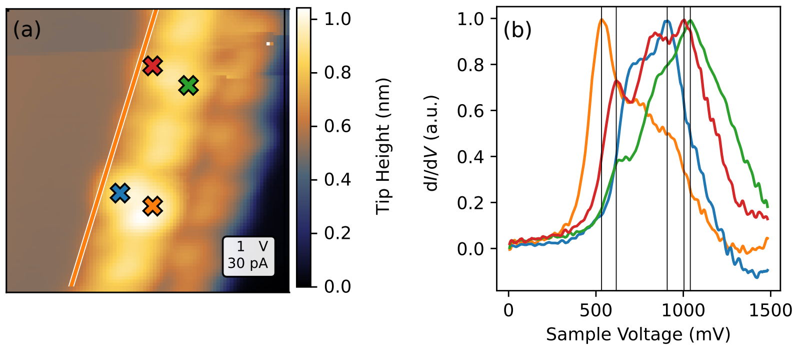

Figure S10: (a) Constant current topograph of a molecular chain containing one A and one A* molecule in the upper row. (b) dI/dV-Spectra acquired at the positions marked in (a) and normalized to identical peak amplitudes. (c) Map of the voltage of the peak in dI/dV extracted from a grid of spectra. The comparison with Figure S9 shows highly reproducible differences between A and A* concerning the energy, spectral shape and spatial distribution of the LUMO resonances.

Energy Diagrams of Tunneling Processes

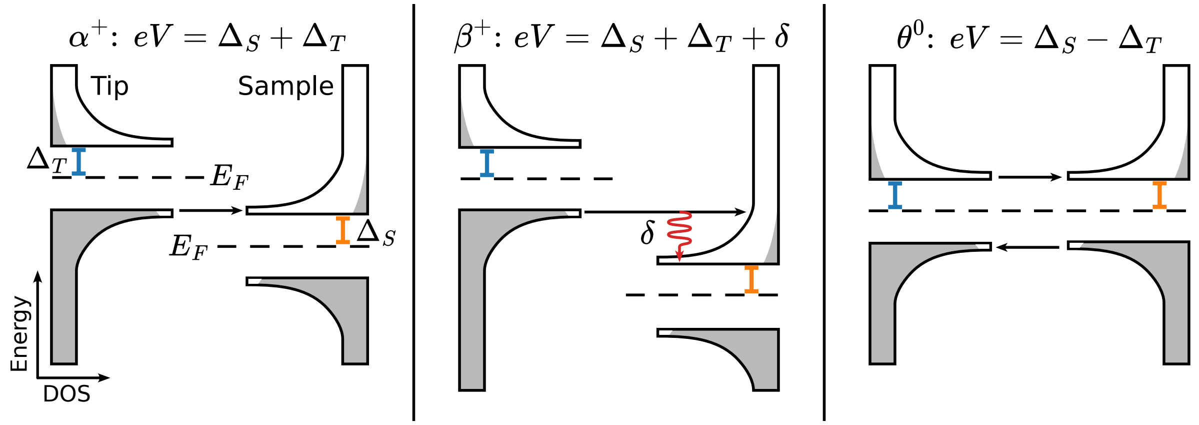

Figure S11: Schematic energy diagrams of tunneling processes (arrows) at characteristic sample voltages $V \geq 0$. The superconducting tip and sample electrodes are represented by their densities of states near the Fermi level (dashed lines). Shading indicates occupations at non-zero temperature (exaggerated for clarity). Coherence peaks α^+ and α^- may be observed at sample voltages $V = \pm 1/e\,(\Delta_T + \Delta_S)$. Spin excitations ($\beta^+$ and β^-, wavy arrows) are efficient at $V = \pm 1/e\,(\Delta_T + \Delta_S + \delta)$. At low bias, $V = \pm 1/e\,(\Delta_S - \Delta_T)$, tunneling of thermally excited quasiparticles (θ^0) may occur.

Fit of Excitation Steps

Most of the spectroscopic data were recorded as current-voltage spectra. Rather than numerically determining dI/dV and fitting temperature broadened steps to the result, we directly used the $I(V)$ data, corrected for current and voltage offsets and fitted matched

integrated step functions as follows:

$$I(V) = a \cdot V + b \cdot V^2 + A \sum_{i=L,U} \frac{-x_{i,+}}{\exp(x_{i,+}) - 1} + \frac{x_{i,-} \exp(x_{i,-})}{\exp(x_{i,-}) - 1}, \qquad (1)$$

where $x_{i,\pm} = (V \pm \delta_i)/k_B T$. A sets the step amplitude and a (b) define a linear (parabolic) background.

Conductance Fits Including Third Order Terms

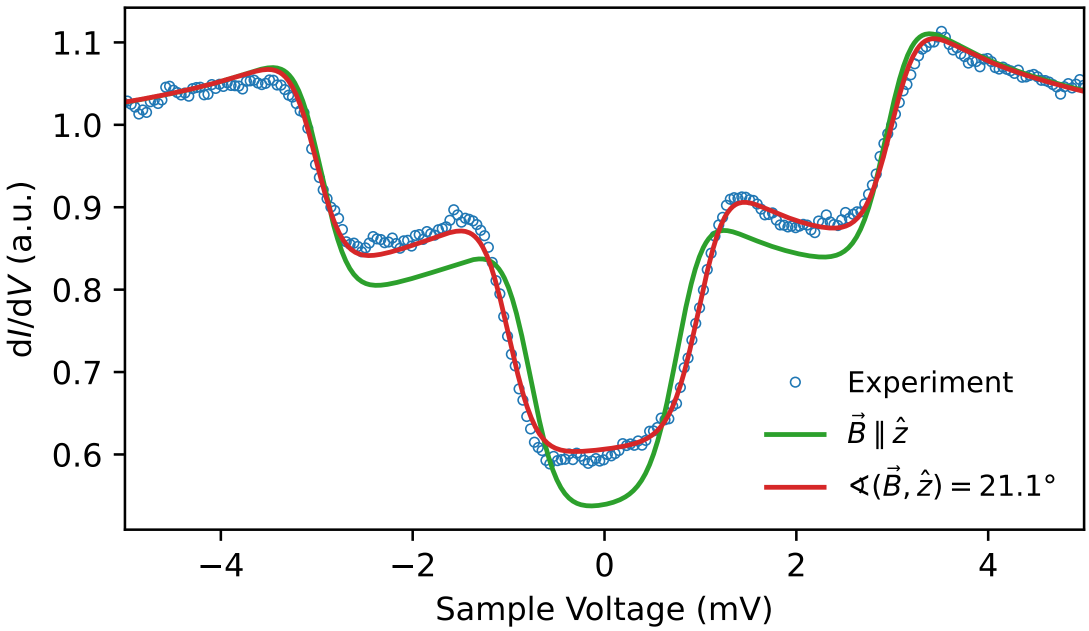

Figure S12: Detailed view of a spectrum recorded at 9 T along with fits that take third order scattering terms into account.[2] The overshoots at the excitation steps are better reproduced. The fit with $\vec{B}$ tilted by 21.1° (red) is clearly superior to the fit neglecting the tilt (green). Experimental data has been acquired using a Lock-in amplifier with $0.5V_{pp}$ modulation at 831 Hz.

Analytical Solutions of the Characteristic Equation

To fit the experimental Zeeman splittings we used the eigenvalues λ_i of the S=1 Hamiltonian $\mathcal{H}$ of Equation 3 of the main article. The characteristic equation $\det(\mathcal{H} - \lambda \mathbb{1}) = 0$ reads

$$\det \begin{pmatrix} D - g\mu_B B_z - \lambda & g\mu_B B_x/\sqrt{2} & 0 \\ g\mu_B B_x/\sqrt{2} & -\lambda & g\mu_B B_x/\sqrt{2} \\ 0 & g\mu_B B_x/\sqrt{2} & D + g\mu_B B_z - \lambda \end{pmatrix} = 0,$$

which leads to

$$\lambda^3 - 2D\lambda^2 + \lambda\left(D - g^2\mu_B^2 B^2\right) + g^2\mu_B^2 D B^2 \sin^2(\phi) = 0$$

For a magnetic field $\vec{B} = (B\sin(\phi),\, 0,\, B\cos(\phi))$ the solutions are

$$\lambda_1 = \frac{2}{3}D - \frac{3B^2g^2\mu_B^2 + D^2}{3Z} - \frac{1}{3}Z$$

$$\lambda_2 = \frac{2}{3}D - \frac{3B^2g^2\mu_B^2 + D^2}{(-1/2 - \sqrt{3}i/2)\cdot 3Z} - (-1/2 - \sqrt{3}i/2)\cdot\frac{1}{3}Z$$

$$\lambda_3 = \frac{2}{3}D - \frac{3B^2g^2\mu_B^2 + D^2}{(-1/2 + \sqrt{3}i/2)\cdot 3Z} - (-1/2 + \sqrt{3}i/2)\cdot\frac{1}{3}Z$$

with

$$Z = (X + Y/2)^{1/3}$$

$$X = 27B^2Dg^2\mu_B^2\sin^2\phi/2 - 8D^3 + 9D\cdot(-B^2g^2\mu_B^2 + D^2)$$

$$Y = \sqrt{-4\cdot(3B^2g^2\mu_B^2 + D^2)^3 + (27B^2Dg^2\mu_B^2\sin^2\phi - 16D^3 + 18D\cdot(-B^2g^2\mu_B^2 + D^2))^2}.$$

These values were fit to the experimental excitation energies: $\lambda_2 - \lambda_1 = \delta_U$ and $\lambda_3 - \lambda_1 = \delta_O$. D, g, and ϕ served as adjustable parameters.

References

1. Gutzeit, F.; Dommaschk, M.; Levin, N.; Buchholz, A.; Schaub, E.; Plass, W.; Näther, C.; Herges, R. Structure and Properties of a Five-Coordinate Nickel(II) Porphyrin. *Inorg. Chem.* **2019**, *58*, 12542–12546.

2. Ternes, M. Spin Excitations and Correlations in Scanning Tunneling Spectroscopy. *New J. Phys.* **2015**, *17*, 063016.
